# Large Spin Hall Conductivity and Excellent Hydrogen Evolution Reaction Activity in Unconventional PtTe_1.75_ Monolayer

**DOI:** 10.34133/research.0042

**Published:** 2023-02-24

**Authors:** Dexi Shao, Junze Deng, Haohao Sheng, Ruihan Zhang, Hongming Weng, Zhong Fang, Xing-Qiu Chen, Yan Sun, Zhijun Wang

**Affiliations:** ^1^ Beijing National Laboratory for Condensed Matter Physics, and Institute of Physics, Chinese Academy of Sciences, Beijing 100190, China.; ^2^ Department of Physics, Hangzhou Normal University, Hangzhou 311121, China.; ^3^ University of Chinese Academy of Sciences, Beijing 100049, China.; ^4^ Shenyang National Laboratory for Materials Science, Institute of Metal Research, Chinese Academy of Science, Shenyang 110016, Liaoning, China.; ^5^ School of Materials Science and Engineering, University of Science and Technology of China, Hefei, China.

## Abstract

Two-dimensional (2D) materials have gained lots of attention due to the potential applications. In this work, we propose that based on first-principles calculations, the (2 × 2) patterned PtTe_2_ monolayer with kagome lattice formed by the well-ordered Te vacancy (PtTe_1.75_) hosts large and tunable spin Hall conductivity (SHC) and excellent hydrogen evolution reaction (HER) activity. The unconventional nature relies on the *A*1 @ 1*b* band representation of the highest valence band without spin–orbit coupling (SOC). The large SHC comes from the Rashba SOC in the noncentrosymmetric structure induced by the Te vacancy. Even though it has a metallic SOC band structure, the ℤ_2_ invariant is well defined because of the existence of the direct bandgap and is computed to be nontrivial. The calculated SHC is as large as 1.25 × 10^3^ $\frac{\hbar }{e}$ (Ω cm)^−1^ at the Fermi level (*E_F_*). By tuning the chemical potential from *E_F_* − 0.3 to *E_F_* + 0.3 eV, it varies rapidly and monotonically from −1.2 × 10^3^ to 3.1 $\times 10^{3}\frac{\hbar }{e}{\left(\Omega \;\mathrm{cm}\right)}^{-1}$. In addition, we also find that the Te vacancy in the patterned monolayer can induce excellent HER activity. Our results not only offer a new idea to search 2D materials with large SHC, i.e., by introducing inversion–symmetry breaking vacancies in large SOC systems, but also provide a feasible system with tunable SHC (by applying gate voltage) and excellent HER activity.

## Introduction

In the past decade, many topological semimetals with various quasiparticle dispersions and fascinating properties have been proposed [1–5]. The layered noble transition metal dichalcogenide PtTe_2_ is extraordinary with heavily tilted type-II Dirac fermion [6]. It hosts unique properties, such as topological nontrivial band structure [6,7], ultrahigh electrical conductivity [8,9], and robustness of the remaining semimetal phase even down to just 2 triatomic layers [10,11]. Soon after, many PtTe_2_ derivatives have been proposed, including the monolayer, multilayer, doping, vacancy, heterojunction structures, and so on. For example, the Ir-doped PtTe_2_ (i.e., Pt_1 − *x*_Ir*_x_*Te_2_) has realized the Fermi level (*E_F_*) tunability and superconductivity, which opens up a new route for the investigation of Dirac physics and topological superconductivity [12–14]. More recently, PtTe_2_-based broadband photodetectors and image sensors have been fabricated, demonstrating tremendous potential application value in various photoelectric devices [15–17]. Very recently, the patterned monolayer with kagome lattice formed by one Te vacancy in a 2 × 2 supercell has been grown successfully [18], whose band topology and potential properties are unknown. The study of PtTe_2_ derivatives can not only reveal novel condensed matter physics but also facilitate the versatile development in device physics.

In this work, we theoretically propose that the recently synthesized patterned PtTe_2_ monolayer with the Te vacancy (i.e., PtTe_1.75_) hosts large spin Hall conductivity (SHC) because of the Rashba spin–orbit coupling (SOC), where the Te vacancy breaks inversion symmetry (I). The momentum offset and strength of the Rashba SOC are estimated, *k*_0_ = 0.12 Å^−1^ and *α_R_* = 0.8112 eV Å. The momentum offset *k*_0_ is very large and comparable with the largest one reported in the Bi/Ag(111) surface alloy in literature [19], which induces visible Rashba band splitting. Using the Kubo formula approach at the clean limit, we find the Rashba SOC will induce large SHC, as large as 1.25 $\times 10^{3}\frac{\mathrm{\hbar \hbar}}{e}{\left(\Omega \;\text{cm}\right)}^{-1}$ at *E_F_*. Furthermore, the SHC changes rapidly and monotonically as the chemical potential evolving in a wide range (*E* − *E_F_* ∈ [−0.3,0.3] eV), which benefits to the potential applications in spintronics. In the end, the variation of the Gibbs free energy for hydrogen adsorption progress is considered, which indicates that the PtTe_1.75_ monolayer exhibit an excellent hydrogen evolution reaction (HER) activity.

## Crystal Structure and Methodology

**Fig. 1.**
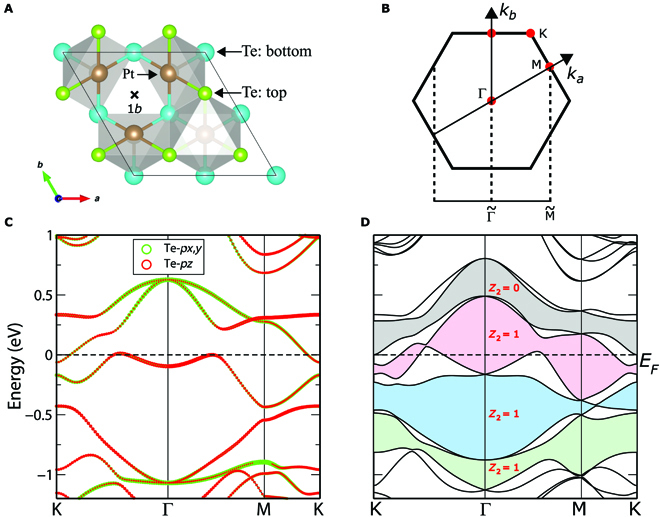
(Color online) (A)Crystal structure of the patterned PtTe_2_ monolayer (PtTe_1.75_). The pristine PtTe_2_ monolayer system in the kagome lattice contains 2 Te layers, with 4 Te atoms in both the bottom layer and the top layer of the (2 × 2) supercell. In addition, the PtTe_1.75_ system comes from the patterned PtTe_2_ monolayer with a well-ordered Te vacancy (schematized by “×” at the 1*b* Wyckoff site) at the top layer of the (2 × 2) supercell. Thus, there are 3 Te atoms (denoted by green ball) occupying the top layer, while there are 4 Te atoms (denoted by blue ball) occupying the bottom layer in the PtTe_1.75_ system. (B) The corresponding 2D bulk BZ and 1-dimensional projected BZ orthogonal to the (01) edge. Band structures of the PtTe_1.75_ system (C) without and (D) with SOC. The light green, blue, red, and gray zones in (D) indicate that there exist direct bandgaps between the corresponding adjacent bands. Thus, the time reversal *ℤ*_2_ can be defined and calculated to be 1, 1, 1, and 0 with (*N_e_* − 4), (*N_e_* − 2), *N_e_*, and (*N_e_* + 2) occupied bands.

The pristine PtTe_2_ crystallizes in the CdI _2_-type trigonal (1*T*) structure with $P\bar{3}m1$ space group (SG). It hosts the layered structure stacking along the *z* axis and can be easily tuned by strain, which indicates that it can be grown under various substrates. The patterned monolayer with kagome lattice formed by one Te vacancy in the 2 × 2 supercell has been successfully grown on the Pt(111) surface [18]. As shown in Fig. 1A, the patterned PtTe_2_ monolayer contains 2 Te layers: 4 Te atoms (blue balls) in the bottom layer and 3 Te atoms (green balls; with one vacancy schematized by “×” at 1*b* Wyckoff site) in the top layer. The distance between the bottom and top layers is *d*_0_ = 2.7253 Å. The Te vacancy breaks I, resulting in a noncentrosymmetrical structure with the p3m1 layer group (LG 69; corresponding to SG *P*3*m*1 excluding translational symmetry along the *z* axis). Thus, the Rashba SOC-induced band splitting is inevitable. The lattice parameters and atomic positions are listed in Table S1 of the Supplementary Materials (SM).

We performed the first-principles calculations based on the density functional theory (DFT) using projector augmented wave method [20,21] implemented in the Vienna ab initio simulation package (VASP) [22,23]. The generalized gradient approximation with exchange–correlation functional of Perdew, Burke, and Ernzerhof for the exchange–correlation functional [24] was adopted. The kinetic energy cutoff was set to 500 eV for the plane wave bases. The thickness of the vacuum layer along *z* axis was set to >20 Å. The BZ was sampled by Γ-centered Monkhorst–Pack method with a 12 × 12 × 1 **k**-mesh for the 2-dimensional (2D) periodic boundary conditions in the self-consistent process. The Wilson loop technique [25] was used to calculate the ℤ_2_ topological invariant. In addition, the electronic structures near *E_F_* were doubly checked by the full-potential local-orbital code [26] and fully consistent with those from VASP. To compute SHC, a Wannier-based tight-binding (TB) model under bases of the Te-*p* and Pt-*d* orbitals is extracted from the DFT calculations.

## Calculations and Results

### Electronic band structures

**Fig. 2.**
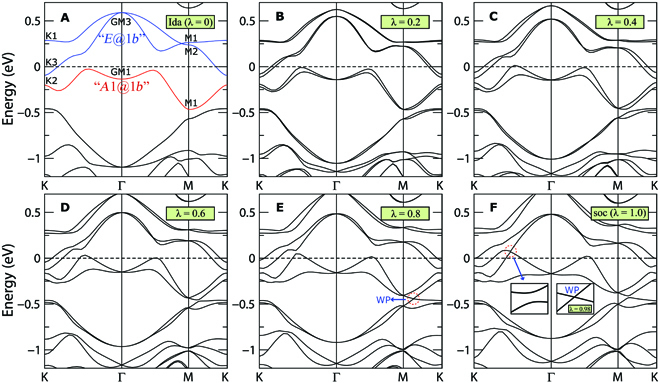
(Color online) Band structures of PtTe_1.75_ monolayer with the strength of SOC (A) *λ* = 0 (without SOC), (B) *λ* = 0.2, (C) *λ* = 0.4, (D) *λ* = 0.6, (E) *λ* = 0.8, and (F) *λ* = 1.0 (with actual SOC). For the case without SOC shown in (A), the 2 conduction bands schematized by 2 blue lines belong to *E* @ 1*b* BR, while the highest valence band schematized by the red line belongs to *A*1 @ 1*b* BR. There exists a WP along the M–K line below *E_F_* when *λ* = 0.8. Bands near *E_F_* undergoes a gap closing and reopening progress when the strength of SOC evolves from 0.0 to 1.0, which gives a topological nontrivial bandgap with SOC (*λ* = 1.0). The critical transition occurs at *λ* = 0.98 (right inset in the *λ* = 1.0 panel), which gives another WP along the K–Γ line.

The band structures of PtTe_1.75_ monolayer without and with SOC are presented in Fig. 1C and D, respectively. Comparing them, we notice that the band dispersions change dramatically. Each band splits into two nondegenerate bands in Fig. 1D. It is the Te vacancy in the monolayer that breaks I (and TI), inducing the visible Rashba band splitting. From the orbital-resolved band structures in Fig. 1C, we find that there exist visible band hybridizations between Te-*p*_*x*,*y*_ and Te-*p_z_* orbitals around *E_F_*. Using IRVSP [27], the irreducible representations of the high-symmetry **k** points are calculated and labeled in Fig. 2A. Accordingly, the band representation (BR) analyses indicate that the 2 conduction bands belong to *E* @ 1*b* BR, while the highest valence band belongs to *A*1 @ 1*b* BR, suggesting the unconventional nature of the obstructed atomic limit [28–31].

SOC often plays important roles to engineering topological states, such as quantum spin Hall effect in graphene [32,33] and Ta_2_M_3_Te_5_ (M = Pd,Ni) compounds [34,35], 3D large-SOC-gap topological insulator in Bi_2_Se_3_ and NaCaBi families [36,37], and so on. In PtTe_1.75_, once including SOC, the Te-*p_z_* dominated band around Γ splits because of the Rashba SOC induced by the Te vacancy, as shown in Fig. 1D. To get more insights in the nontrivial band topology and Rashba SOC band splitting, we have explored how the band structure evolves with the increasing strength of SOC (denoted by *λ*) gradually in Fig. 2. We notice that the nontrivial band topology for *N_e_* − 4 occupied bands is due to the SOC (can be infinitesimal)-induced bandgap at Γ without involving band inversion [38,39]. In addition, the nontrivial topologies for *N_e_* − 2 and *N_e_* occupied bands are due to a gap closing and reopening process as varing *λ*. Taking *N_e_* − 2 occupied bands as an example, the critical Weyl band crossing between the (*N_e_* − 2)th band and the (*N_e_* − 1)th band appears on the M–K line with *λ* = 0.8, as highlighted by a red dashed ring in Fig. 2E. Similarly, the critical Weyl point (WP) between the *N_e_*th band and the (*N_e_* + 1)th band appears on the K–Γ line with *λ* = 0.98, as the right inset shown in Fig. 2F. However, it becomes topologically trivial for *N_e_* + 2 occupied bands because there are 2 nontrivial gap openings around both Γ and M.

**Fig. 3.**
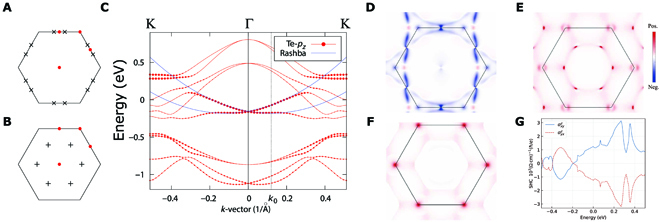
(Color online) The 6 symmetry-related (*R*_3*z*_ and *M*_100_) WPs formed by band crossings (A) between the (*N_e_* − 2)th band and the (*N_e_* − 1)th band with *λ* = 0.8 as well as (B) the *N_e_*th band and the (*N_e_* + 1)th band with *λ* = 0.98. (C) The Te-*p_z_* projected band structures with SOC, and the 2 blue lines depict the parabolically asymptotic behavior of the Rashba SOC induced splitting bands near the Γ point. The distribution of (D) $O_{N_{e}-1}\left(k\right)$ and (E) $O_{N_{e}}\left(k\right)$ in the 2D BZ. (F) The distribution of $\sigma_{\mathrm{xy}}^{z}$ at *E_F_*. (G) The calculated SHC vs. the chemical potential (ranging from *E_F_* − 0.5 eV to *E_F_* + 0.5 eV).

Because of the existence of *R*_3*z*_ and *M*_100_ symmetries, the critical WPs abovementioned appear in sextuplet in the first BZ, as shown in Fig. 3A and B. Similar with WPs in 3D Weyl semimetals [40–43], these critical WPs also conform to the codimensional analysis. This can be deduced as follows. First, both the M–K (*k_y_* = *π*) and K–Γ (*k_y_* = 0) lines are *M*_100_ ∗ *T* invariant. In the 2-band ***k*** · ***p*** Hamiltonian depicting the Weyl band crossing, the combined antiunitary symmetry with [*TM*_100_]^2^ = 1 will reduce the number of the independent *σ* matrices in the ***k*** · ***p*** Hamiltonian to 2. Second, the *k_x_* value in both the *TM*_100_ invariant lines and the SOC strength *λ* are 2 tunable parameters to search a WP. Thus, the number of the independent *σ* matrices in the ***k*** · ***p*** Hamiltonian equals to the number of the tunable parameters, which indicates that a WP is stable in the 2D parameter space {*k_x_*, *λ*}. In other words, a topological phase transition can happen by tuning *λ* in the *M*_100_ ∗ *T* invariant lines. Through the gap closing and reopening process in the evolution, it becomes topologically nontrivial for *N_e_* (*N_e_* − 2) occupied bands. As a result, we can expect the existence of the helical edge states of the patterned PtTe_2_ monolayer. The edge spectra are presented in Fig. S3B and D (in Section C of the SM).

### Rashba SOC at Γ

Because the Te vacancy breaks I in the patterned PtTe_2_ monolayer, the Rashba SOC band splitting will appear inevitably. As the projected band structures shown in Fig. 3C, the Te-*p_z_* dominated parabolic bands splits clearly near Γ. The splitting bands near Γ can be well fitted by $E=\frac{{\left[\mathrm{\hbar \hbar}\left(k\pm k_{0}\right)\right]}^{2}}{2M}$ with *M* = 2.02659 *m_e_* (*m_e_* denoting the free electronic mass) and *k*_0_ = 0.12 Å^−1^ (the momentum offset), as the 2 blue parabolas shown in Fig. 3C. The coupling strength of the Rashba SOC can be derived as $\alpha_{R}=\frac{2E_{R}}{k_{0}}=\frac{{\mathrm{\hbar \hbar}}^{2}k_{0}}{M}=0.81$ eVÅ. The estimated *k*_0_ is super large in Fig. 3C, as large as the the Bi/Ag(111) surface alloy [19].

### Large SHC effect

To explore the intrinsic SHC in the patterned PtTe_2_ monolayer, the Wannier-based TB model under bases of the Te-*p* and Pt-*d* orbitals is extracted from the DFT calculations. As shown in Fig. S2A and B, the fitted Wannier-based TB bands can reproduce the DFT bands perfectly. On the basis of this Wannier-based TB model, we have employed the Kubo formula approach at the clean limit [44–48] to calculate the SHC of the patterned PtTe_2_ monolayer,

$$\begin{array}{c} \sigma_{\alpha \beta }^{\gamma }=\frac{e}{\hbar }\sum_{n}‍\int_{\mathrm{BZ}}‍\frac{dk}{{\left(2\pi \right)}^{2}}f_{n}\left(k\right)\Omega_{\alpha \beta ;n}^{\gamma }\left(k\right),\\ \Omega_{\alpha \beta ;n}^{\gamma }\left(k\right)=2i\hbar^{2}\sum_{m\neq n}\frac{\langle u_{k}^{n}|{\hat{J}}_{\alpha }^{\gamma }|u_{k}^{m}\rangle \langle u_{k}^{m}|{\hat{v}}_{\beta }|u_{k}^{n}\rangle }{{\left(\varepsilon_{k}^{n}-\varepsilon_{k}^{m}\right)}^{2}}, \end{array}$$ (1)

where ${\hat{J}}_{\alpha }^{\gamma }=\frac{1}{2}\left\{{\hat{v}}_{\alpha },{\hat{s}}_{\gamma }\right\}$ is the spin current operator, with $\hat{s}$ denoting the spin operator, ${\hat{v}}_{\alpha }=\frac{\partial \hat{H}}{\mathrm{\hbar \hbar}\partial k_{\alpha }}$ denoting the velocity operator, and *α*, *β*, *γ* = {*x*, *y*, *z*}. *f_n_*(**k**) is the Fermi–Dirac distribution. $|u_{k}^{n}\rangle$ and $\varepsilon_{k}^{n}$ are the eigenvectors and eigenvalues of the TB Hamiltonian, respectively. The distributions of $O_{N}\left(k\right)\equiv \sum_{n=1}^{N}‍\Omega_{\mathrm{yz};n}^{x}\left(k\right)$ for *N* = *N_e_* − 1 and *N_e_* occupied bands are presented in Fig. 3D and E, respectively. As the calculated SHC as a function of the chemical potential shown in Fig. 3G, one can find that the calculated SHC is as large as 1.25 $\times 10^{3}\frac{\mathrm{\hbar \hbar}}{e}{\left(\Omega \;\text{cm}\right)}^{-1}$ at *E* = *E_F_*. The corresponding distribution at *E* = *E_F_* is presented in Fig. 3F, which indicates that the large contribution of the SHC at K comes from the SOC band splitting. In addition, the SHC changes rapidly and monotonically in a wide energy window ranging from *E_F_* − 0.3 eV to *E_F_* + 0.3 eV. At *E* − *E_F_* = −0.3 eV, the SHC changes the sign and becomes $-\;1.2\times 10^{3}\frac{\hbar }{e}{\left(\Omega \;\text{cm}\right)}^{-1}$, while at *E* − *E_F_* = 0.3 eV, the SHC nearly triples and becomes 3.1 $\times 10^{3}\frac{\mathrm{\hbar \hbar}}{e}{\left(\Omega \;\text{cm}\right)}^{-1}$. In general, the chemical potential can be tuned by applying gate voltage or introducing chemical doping at the vacancy. As shown in Fig. S5A to C, we can find that the absorption of Tl/Pb at the vacancy behaves as electron dopings, which will increase the *E_F_* with negligible changes in the band structure. We think our results will be benefitial to the potential applications in spintronics.

### Excellent HER activity

**Fig. 4.**
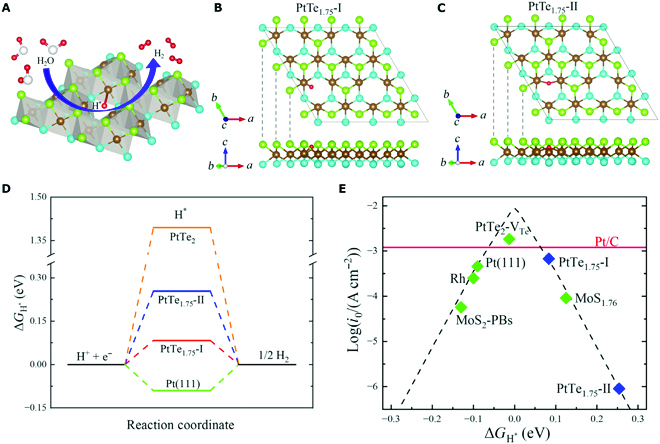
(Color online) (A) Schematic diagram of HER process on PtTe_1.75_ monolayer. Top and side views of (B) the most stable (PtTe_1.75_-I) and (C) metastable (PtTe_1.75_-II) structures after H atom adsorption. The red ball denotes the absorbed H atom. (D) Variation of the Gibbs free energy for hydrogen adsorption (Δ*G*_H^∗^_) to different compounds. (E) Volcano plot depicting the relationship between exchange current density (*i*_0_) and Δ*G*_H^∗^_, in which cases of Pt(111) [53], Rh [53], Pt/C [57], PtTe_2_ with ordered trigonal Te vacancies (PtTe_2_-V_Te_) [51], MoS_1.76_ [52], and 2H-1T phase boundaries of MoS_2_ (MoS_2_-PBs) [58] are also included for comparison.

According to the new principle for active catalytic sites [30,49,50], the obstructed bulk states in the patterned monolayer (which can be seen as the limit of obstructed surface states) may bring measured catalytic activity. By exposing undercoordinated atoms as the active sites, vacancy engineering is an important strategy to optimize the HER performance of the basal planes in 2D materials [51,52]. As the acidic HER of the PtTe_1.75_ is schematized in Fig. 4A, protons (H^+^) in solution generate adsorbed H atoms (H^∗^) as intermediate and then the H atoms on the catalyst surface are desorbed to produce hydrogen (H_2_), which can be formulized as

$$H^{+}+e^{-}+*\to H^{*}\to \frac{1}{2}H_{2}+*.$$ (2)

Here “*” denotes some site on the surface, i.e., a “*” by itself denotes a free site, while H* denotes a hydrogen atom absorbed on the surface. Te vacancy-induced states near *E_F_* give PtTe_1.75_ monolayer larger electrical conductivity than pristine PtTe_2_ monolayer, which will effectively facilitate electron transfer for HER. We used a 2 × 2 PtTe_1.75_ supercell to simulate the basal plane. Compared with the fully coordinated Te atoms, H atoms are more likely to be adsorbed near the undercoordinated Pt atoms, just as the most stable and metastable structures shown in Fig. 4B and C. Details of screening stable adsorption sites can be found in the SM. It is well known that the change of Gibbs free energy induced by hydrogen adsorption (Δ*G*_H^∗^_) is an important descriptor of HER activity [53–55], and an ideal catalyst for HER should host a near-zero Δ*G*_H^∗^_, which can effectively maintain the balance between adsorption and desorption steps [54]. As shown in Fig. 4D, unlike the pristine PtTe_2_ monolayer with a large positive Δ*G*_H^∗^_ due to its extremely inert basal plane, the PtTe_1.75_ monolayer hosts an optimal Δ*G*_H^∗^_ (0.08 eV), which is slightly superior to the benchmark material Pt (Δ*G*_H^∗^_ = −0.09 eV) [53]. Details of the free energy correction can be found in the SM. Here, we noted that the effect of the size on the Δ*G*_H^∗^_ is negligible, which can be deduced from Table S2. Thus, the active Pt sites induced by Te vacancy greatly optimize hydrogen adsorption in the intermediate, which will significantly improve HER performance [56]. According to Nørskov *et al.* [53], the theoretical exchange current density (*i*_0_) as a function of Δ*G*_H^∗^_ is calculated. As shown in Fig. 4E, the PtTe_1.75_ monolayer approaches the volcanic peak from the right with *i*_0_ = 0.68 mA cm^−2^, which is comparable to commercial Pt/C catalyst (*i*_0_ = 1.2 mA cm^−2^) [57]. In addition, as shown in Fig. S4A and B, the energy pathways and corresponding energy barriers of the (a) Heyrovsky and (b) Tafel reactions to release hydrogen in HER are exhibited, from which we can find that the Tafel reactions to release hydrogen is preferred. Therefore, Te vacancy can greatly stimulate the catalytic activity of PtTe_2_ basal plane and produce excellent HER performance.

## Discussion

We find that the PtTe_1.75_ not only hosts the unique band structure with 3 lower-energy bands belonging to (*A*1 + *E*) @ 1*b* BRs at an empty site but also exhibits large and tunable SHC and excellent HER performance. First, we have calculated the time reversal invariant ℤ_2_, which indicates the 2D TI nature in the patterned PtTe_2_ monolayer. We demonstrate that the topological phase can be deduced by a gap closing and reopening process with the evolution of the strength of SOC from *λ* = 0 to *λ* = 1.0. The critical phase transition occurs at *λ* = 0.98, which gives a sextuplet of critical WPs. Second, the Te vacancy breaks I and induces Rashba SOC band splitting. The estimated momentum offset is super large with *k*_0_ = 0.12 Å^−1^. Third, we find that the SHC is as large as 1.25 $\times 10^{3}\frac{\mathrm{\hbar \hbar}}{e}{\left(\Omega \;\text{cm}\right)}^{-1}$ at *E_F_*. Furthermore, the SHC varies quickly and almost monotonically from −1.2 to 3.1 $\times 10^{3}\frac{\mathrm{\hbar \hbar}}{e}{\left(\Omega \;\text{cm}\right)}^{-1}$, indicating that the SHC in the patterned PtTe_2_ monolayer can be conveniently tuned for various applications. Last, we also find the Te vacancy in the patterned monolayer can induce excellent HER activity. These results not only offer a new idea to search 2D materials with large SHC, i.e., by introducing inversion–symmetry breaking vacancies in large SOC systems, but also provide a feasible system for the potential application in spintronics and HER catalysts.

## Data Availability

The datasets used in this article are available from the corresponding author upon request.

## Supplementary Materials

Supplementary Materials Section A. Lattice parameters of the patterned PtTe_2_ monolayer with a Te vacancy. Section B. The calculated weak topological invariant Z_2_. Section C. Topological surface states of the patterned PtTe_2_ monolayer. Section D. Releasing hydrogen in HER. Section E. Screening adsorption sites and the correction of Gibbs free energy. Section F. Band structures vs. dopping. Table S1. Crystal structures of the patterned PtTe_2_ monolayer in terms of SG *P*3*m*1 (SG 156). Table S2. The Gibbs free energy correction terms of the most thermodynamically stable PtTe_1.75_ adsorption structures with 1 × 1, 2 × 2, 3 × 3, and 4 × 4 supercell, including adsorption energies of hydrogen (Δ*E_H_**), the change of zero-point energy (ΔZPE), enthalpy correction (Δ∫*C_p_*dT), entropy correction (ΔTS), and Gibbs free energy (ΔG_H_*). Fig. S1. (Color online) The calculated weak topological invariant Z_2_ for (A) 80, (B) 82, (C) 84, and (D) 86 occupied bands, respectively. Fig. S2. (Color online) DFT vs. Wannier bands (A) without SOC and (B) with SOC. Fig. S3. (Color online) The projected edge states along (01) direction (A) without SOC and (B) with SOC in the upper edge. Fig. S4. (Color online) Energy pathways of the (A) Heyrovsky and (B) Tafel reactions on PtTe_1.75_ monolayer to release hydrogen. Fig. S5. (Color online) Band structures of the patterned PtTe_2_ monolayer with (A) no doping (Te vacancy), (B) Tl doping, and (C) Pb doping at the Te vacancy position. References [59–62]
