# Supplementary material for: Large Spin Hall Conductivity and Excellent Hydrogen Evolution Reaction Activity in Unconventional PtTe1.75 Monolayer
Source: Research (Wash D C). 2023 Feb 24;6:0042. doi: 10.34133/research.0042 (PMC10013811; doi:10.34133/research.0042)
Supplement: Supplementary Materials — Section A. Lattice parameters of the patterned PtTe2 monolayer with a Te vacancy. Section B. The calculated weak topological invariant Z2. Section C. Topological surface states of the patterned PtTe2 monolayer. Section D. Releasing hydrogen in HER. Section E. Screening adsorption sites and the correction of Gibbs free energy. Section F. Band structures vs. dopping. Table S1. Crystal structures of the patterned PtTe2 monolayer in terms of SG P3m1 (SG 156). Table S2. The Gibbs free energy correction terms of the most thermodynamically stable PtTe1.75 adsorption structures with 1 × 1, 2 × 2, 3 × 3, and 4 × 4 supercell, including adsorption energies of hydrogen (ΔEH*), the change of zero-point energy (ΔZPE), enthalpy correction (Δ∫CpdT), entropy correction (ΔTS), and Gibbs free energy (ΔGH*). Fig. S1. (Color online) The calculated weak topological invariant Z2 for (A) 80, (B) 82, (C) 84, and (D) 86 occupied bands, respectively. Fig. S2. (Color online) DFT vs. Wannier bands (A) without SOC and (B) with SOC. Fig. S3. (Color online) The projected edge states along (01) direction (A) without SOC and (B) with SOC in the upper edge. Fig. S4. (Color online) Energy pathways of the (A) Heyrovsky and (B) Tafel reactions on PtTe1.75 monolayer to release hydrogen. Fig. S5. (Color online) Band structures of the patterned PtTe2 monolayer with (A) no doping (Te vacancy), (B) Tl doping, and (C) Pb doping at the Te vacancy position. References [59–62] [file research.0042.f1.pdf]

SUPPLEMENTARY MATERIAL

A. Lattice parameters of the patterned $PtTe_2$ monolayer with a Te vacancy

The pristine $PtTe_2$ system crystallizes in the CdI_2-type trigonal (1T) structure (SG P$\bar{3}$m1) is a layered material stacking along the z axis. Recently, the monolayer structure with kagome lattice formed by one Te vacancy in the 2×2 supercell has been successfully grown[18]. The patterned $PtTe_2$ monolayer contains two Te layers, with 4 Te atoms in the bottom layer while 3 Te atoms in the top layer, as shown in Fig. 2(a). The corresponding lattice parameters are $a = b = 8.1846\mathring{A}$, $\alpha = \beta = 120°$, and the thickness of the vacuum layer along z axis was set to $30 - d_0$ Å, with d_0 ($= 2.7253\mathring{A}$) denoting the distance between the bottom Te layer and the top Te layer.

TABLE S1. Crystal structures of the patterned $PtTe_2$ monolayer in terms of space group P3m1 (SG 156).

Atoms	Wyckoff positions	Fractional coordinates
Pt1	1c	(0.66667,0.33333,0.0)
Pt2	3d	(0.16667,0.33333,0.0)
Te1(bottom)	1a	(0.00000,0.00000,-0.04542)
Te2(bottom)	3d	(0.50000,0.50000,-0.04542)
Te3(top)	3d	(0.83333,0.66667,0.04542)

B. The calculated weak topological invariant Z_2

To characterize the topological properties in the patterned $PtTe_2$ monolayer, the weak topological invariants are calculated by the 1D Wilson loop method. Taking Te: $5s^25p^4$ and Pt: $5d^96s^1$ orbitals into consideration, there are $N_e = 82$ valance electrons, resulting in N_e valence bands. From Figs. S1(a-d), the calculated weak topological invariants for 78, 80, 82 and 84 (corresponds to $N_e - 4$, $N_e - 2$, N_e and $N_e + 2$) occupied bands are $\mathbb{Z}_2 = 1$, $\mathbb{Z}_2 = 1$, $\mathbb{Z}_2 = 1$ and $\mathbb{Z}_2 = 0$, respectively. Thus, the patterned $PtTe_2$ monolayer with N_e valence bands is a 2D TI with $\mathbb{Z}_2 = 1$.

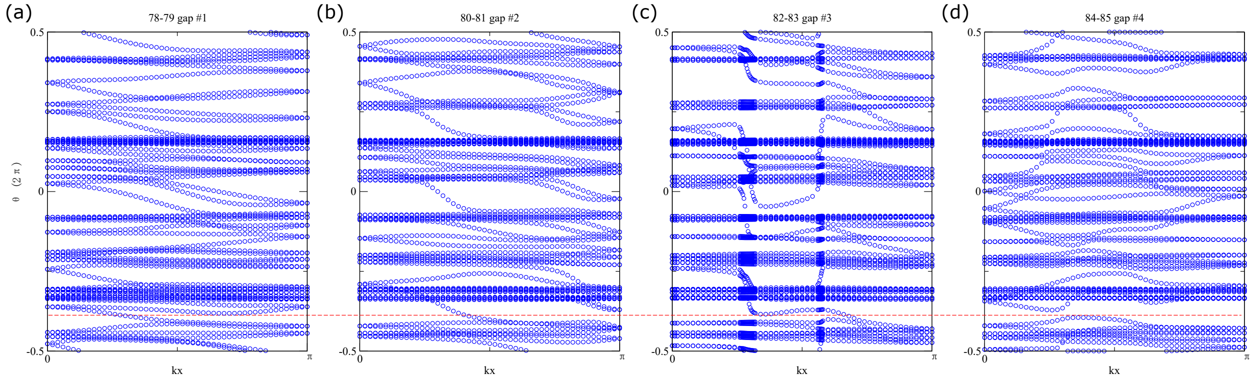

FIG. S1. (Color online) The calculated weak topological invariant $\mathbb{Z}_2$ for (a) 80 (b) 82 (c) 84 and (86) occupied bands, respectively.

C. Topological surface states of the patterned $PtTe_2$ monolayer

Exotic topological surface states serve as significant fingerprints to identify various topological phases. Based on the tight-binding (TB) model constructed with the maximally localised Wannier functions and surface Green function methods[59–61], we have calculated the corresponding surface states to identify the nature of 2D TI.

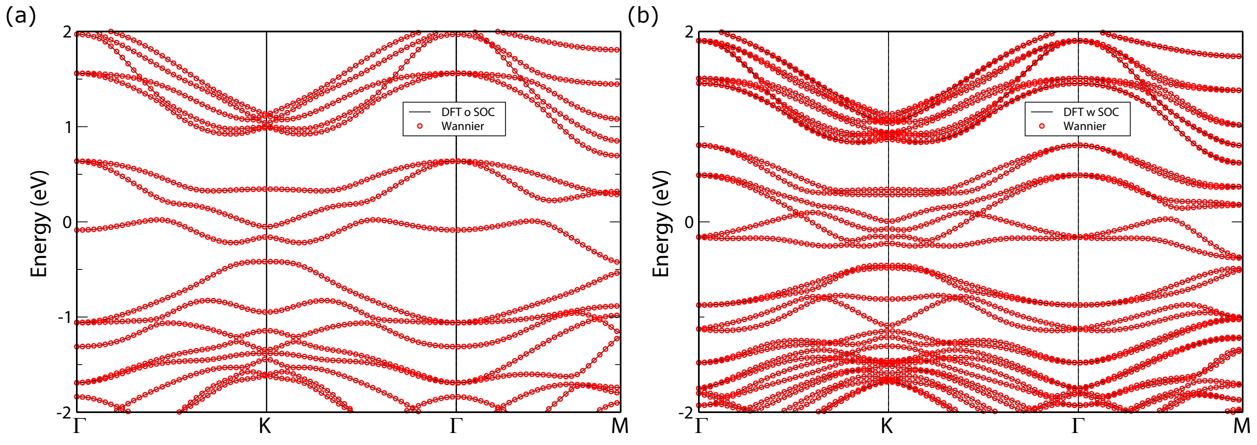

FIG. S2. (Color online) DFT vs. Wannier bands (a) without SOC and (b) with SOC.

As shown in Fig.S2(a,b), we have chosen Pt-d and Te-p orbitals as the projected bases of the wannier-based TB model, which can reproduce the DFT band structures perfectly. As expected, the helical edge states corresponds to the 2D TI can be found in the (01) edge states, as shown in Fig. S3(b) and Fig. S3(d).

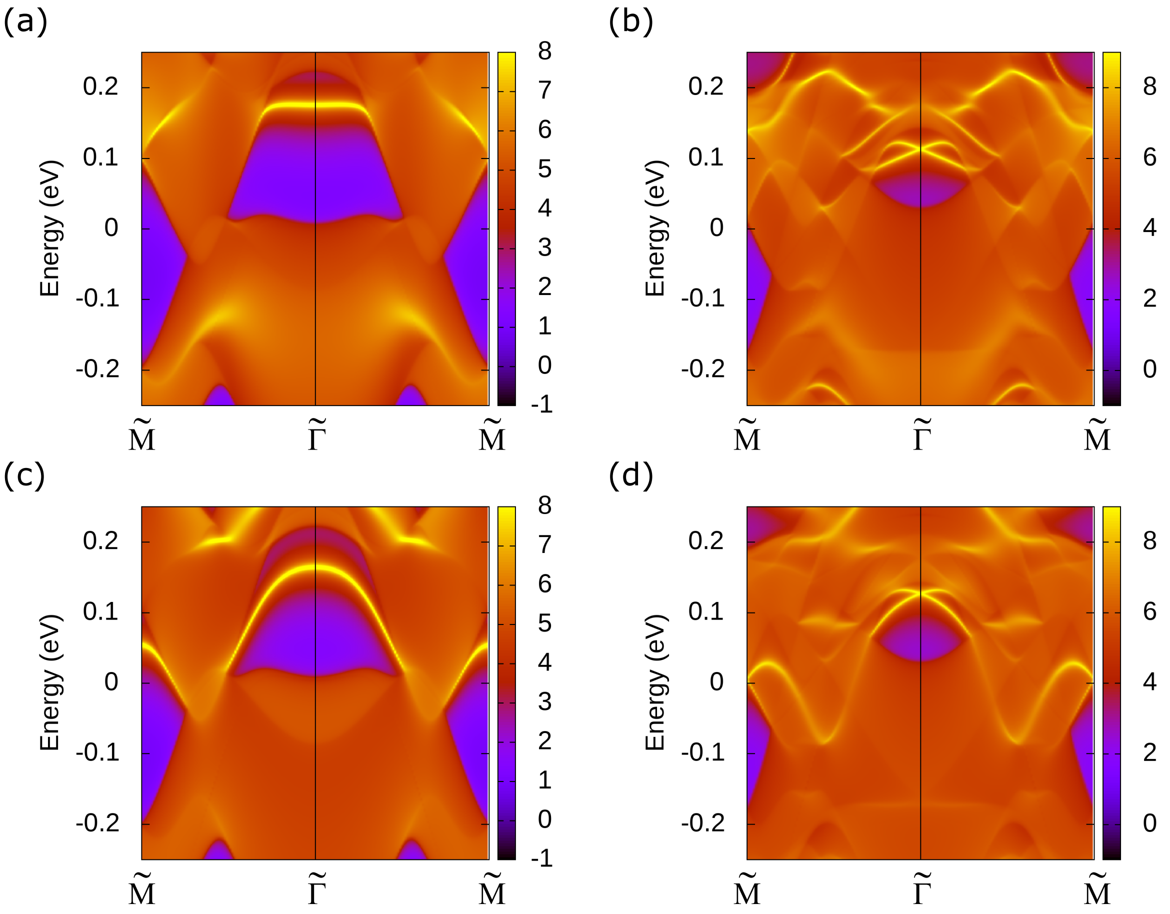

FIG. S3. (Color online) The projected edge states along (01) direction (a) without SOC and (b) with SOC in the upper edge. The projected edge states along (01) direction (c) without SOC and (d) with SOC in the lower edge.

D. Releasing hydrogen in hydrogen evolution reaction

There are two different mechanisms of the releasing hydrogen in HER. One is achieved by combining a solvated proton with an adsorbed H atom $H^+ + e^- + H^* \rightarrow H_2$, known as Heyrovsky reaction, while the other one is achieved by combining two adsorbed H atoms $2H^* \rightarrow H_2$, i.e., the Tafel reaction. As shown in Fig. S4, the energy barrier in the Tafel reaction is assessed with a smaller value (0.87 eV) by the climbing-image nudged elastic band (CI-NEB) approach[62], which is 0.57 eV lower than that in the Heyrovsky reaction.

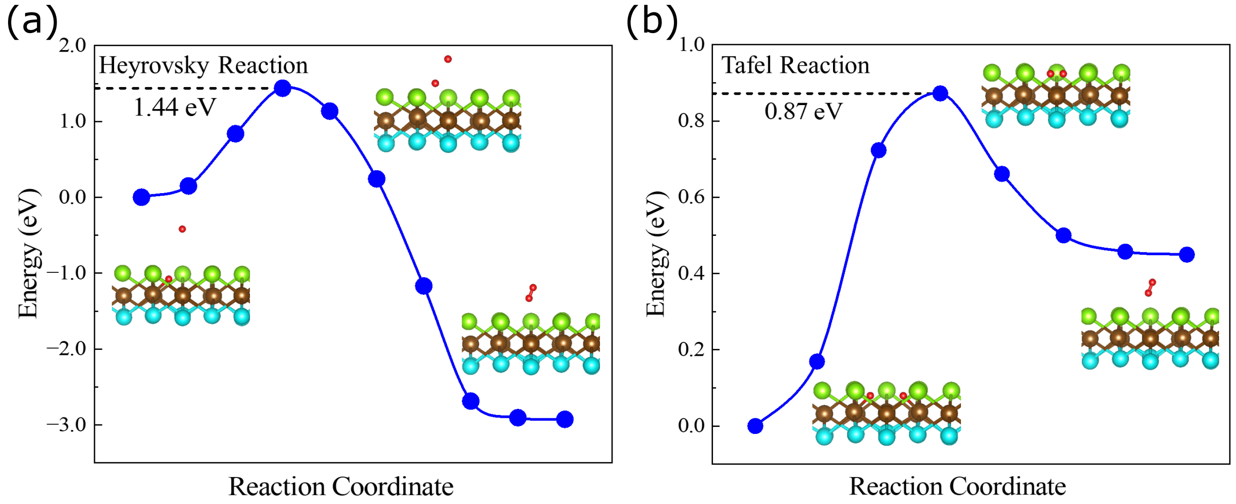

FIG. S4. (Color online) Energy pathways of the (a) Heyrovsky and (b) Tafel reactions on $PtTe_{1.75}$ monolayer to release hydrogen. The insets are the side view of the initial, transition and final states.

E. Screening adsorption sites and the correction of Gibbs free energy

In order to screen multiple thermodynamically stable adsorption sites, we uniformly generated 100 hydrogen adsorption structures with the {x,y} coordinates of the H atoms dispersing uniformly in the quarter of the 2×2 $PtTe_{1.75}$ supercell. After optimizing the z coordinate of the adsorbed H atoms, we choose five most thermodynamically stable structures. After the fully structural optimization, we find that four of them host the same ultimate configuration (denoted as $PtTe_{1.75}$-I) with the lowest energy. While the other ultimate configuration (denoted as $PtTe_{1.75}$-II) is metastable with a 6.14 meV/atom higher energy than the $PtTe_{1.75}$-I phase.

The change of Gibbs free energy induced by hydrogen adsorption (ΔG_{H^*}) can be defined as[55]

$$\begin{aligned} \Delta G_{H^*} &= G(H^*) - G(*) - \frac{1}{2}G(H_2) \\ G &= E + ZPE + \int C_p dT - TS \end{aligned} \tag{3}$$

where E is internal Energy, ZPE is zero-point energy, $\int C_p dT$ is the correction of enthalpy, T is temperature, while S denotes entropy. For the adsorption structure (H*), we only pay attention to the vibration contribution of the adsorbed H atom, while the correction energy of the whole $PtTe_{1.75}$ slab can be considered as unchanged before and after the absorbed process. In order to take the effect of structural size on ΔG_{H^*} into consideration, we have chosen the most thermodynamically stable PtTe1.75 adsorption structures with 1×1, 2×2, 3×3 and 4×4 supercell. As shown in Table S2, we can find that the effect of the size is almost negligible.

F. Band structures vs. dopping

Since the electronic properties and SHC of the patterned $PtTe_2$ monolayer are sensitive to the E_F, we can adopt different dopings at the Te-vacancy position to tune E_F effectively.

TABLE S2. The Gibbs free energy correction terms of the most thermodynamically stable $PtTe_{1.75}$ adsorption structures with 1×1, 2 × 2, 3 × 3 and 4 × 4 supercell, including adsorption energies of hydrogen (ΔE_{H^*}), the change of zero-point energy (ΔZPE), enthalpy correction ($\Delta \int C_p$ dT), entropy correction (ΔTS) , and Gibbs free energy (Δ GH*). All units are eV.

	ΔE_{H^*}	ΔZPE	$\Delta \int C_p dT$	ΔTS	ΔG_{H^*}
1×1	-0.1464	0.0732	-0.0371	-0.1911	0.0809
2×2	-0.1438	0.0725	-0.0369	-0.1909	0.0826
3×3	-0.1401	0.0734	-0.0371	-0.1911	0.0874
4×4	-0.1471	0.0734	-0.0370	-0.1911	0.0803

As shown in Figs. S5(a-c), we can find that both the introduced Pb doping and Tl doping behave as electron dopings, which increase the E_F significantly. The decreased magnitude of the E_F are estimated to be 0.482232 eV and 0.256149 eV, respectively.

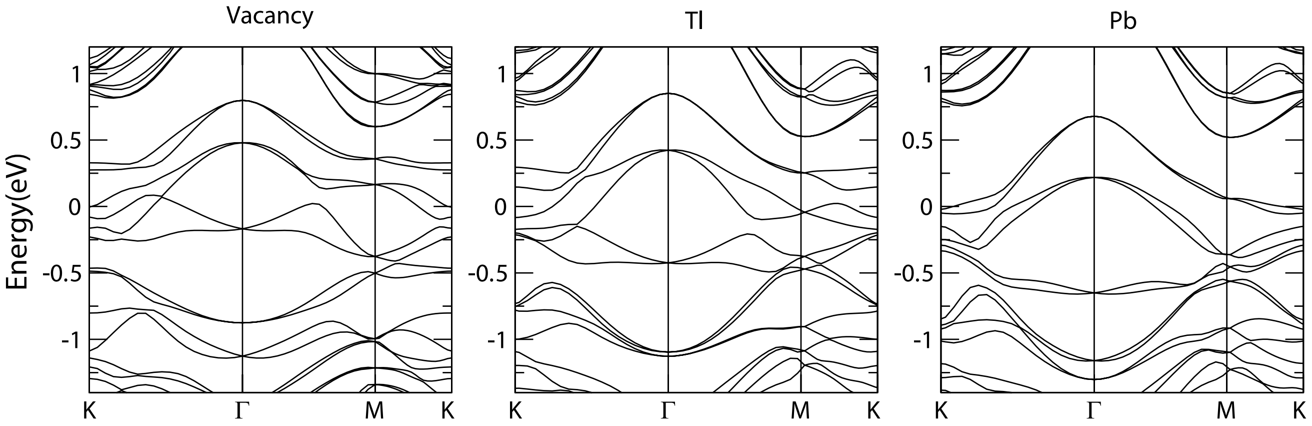

FIG. S5. (Color online) Band structures of the patterned $PtTe_2$ monolayer with (a) no doping (Te vacancy), (b) Tl doping, and (c) Pb doping at the Te-vacancy position.
